# Alien insect dispersal mediated by the global movement of commodities

**DOI:** 10.1002/eap.2721

**Published:** 2022-11-13

**Authors:** Gyda Fenn‐Moltu, Sébastien Ollier, Barney Caton, Andrew M. Liebhold, Helen Nahrung, Deepa S. Pureswaran, Rebecca M. Turner, Takehiko Yamanaka, Cleo Bertelsmeier

**Affiliations:** ^1^ Department of Ecology and Evolution University of Lausanne Lausanne Switzerland; ^2^ Department of Ecology, Systematics and Evolution University Paris‐Saclay Orsay France; ^3^ United States Department of Agriculture, Animal and Plant Health Inspection Services Plant Protection and Quarantine Raleigh North Carolina USA; ^4^ USDA Forest Service Northern Research Station Morgantown West Virginia USA; ^5^ Faculty of Forestry and Wood Sciences Czech University of Life Sciences Prague Suchdol Czech Republic; ^6^ Forest Research Institute University of the Sunshine Coast Maroochydore DC Queensland Australia; ^7^ Canadian Forest Service Laurentian Forestry Centre Quebec City Quebec Canada; ^8^ Scion (New Zealand Forest Research Institute) Christchurch New Zealand; ^9^ Institute for Agro‐Environmental Sciences, NARO Tsukuba Japan

**Keywords:** commodity trade, globalization, human‐mediated dispersal, insects, introduction pathways, invasion risk

## Abstract

Globalization and economic growth are recognized as key drivers of biological invasions. Alien species have become a feature of almost every biological community worldwide, and rates of new introductions continue to rise as the movement of people and goods accelerates. Insects are among the most numerous and problematic alien organisms, and are mainly introduced unintentionally with imported cargo or arriving passengers. However, the processes occurring prior to insect introductions remain poorly understood. We used a unique dataset of 1,902,392 border interception records from inspections at air, land, and maritime ports in Australia, New Zealand, Europe, Japan, USA, and Canada to identify key commodities associated with insect movement through trade and travel. In total, 8939 species were intercepted, and commodity association data were available for 1242 species recorded between 1960 and 2019. We used rarefaction and extrapolation methods to estimate the total species richness and diversity associated with different commodity types. Plant and wood products were the main commodities associated with insect movement across cargo, passenger baggage, and international mail. Furthermore, certain species were mainly associated with specific commodities within these, and other broad categories. More closely related species tended to share similar commodity associations, but this occurred largely at the genus level rather than within orders or families. These similarities within genera can potentially inform pathway management of new alien species. Combining interception records across regions provides a unique window into the unintentional movement of insects, and provides valuable information on establishment risks associated with different commodity types and pathways.

## INTRODUCTION

The globalization of human activities facilitates species dispersal across historical biogeographic barriers, such that alien species are now an established part of almost every biological community worldwide (Convention on Biological Diversity, 2001). As the international movement of people and goods accelerates and expands, the rate of new introductions continues to rise (Levine & D'Antonio, 2003; Seebens et al., 2017). Some species that are introduced and overcome biotic and abiotic barriers to establishment (Blackburn et al., 2011) cause harmful ecological or economic impacts in their new range (Pagad et al., 2015). In terrestrial ecosystems, insects are among the most numerous and problematic alien organisms, costing at least US$70 billion per year globally (Bradshaw et al., 2016; Diagne et al., 2021). Unlike most alien vertebrates and plants, insects are usually introduced unintentionally (Rabitsch, 2010). This typically occurs through the transport of commodities, either because the commodity is their natural host or their immediate environment (contaminant pathway), or because insects have actively attached to an object not directly related to their natural environment (hitchhiking pathway) (Gippet et al., 2019). Introduction pathways encompass the suite of processes that transport a species from one location to another, including both the vector and the human activity resulting in an introduction (Genovesi & Shine, 2004; Pyšek et al., 2011).

Managing introduction pathways and corresponding commodities is therefore a potentially powerful strategy for preventing new introductions, and thereby reducing negative impacts on biodiversity, human health (Mazza et al., 2014; Pratt et al., 2017; Pyšek & Richardson, 2010) and economies (Bradshaw et al., 2016). Risk assessment strategies for alien species often prioritize identifying sources and pathways of introduction (Hulme et al., 2008). Yet when assessing establishment risks and mitigation measures, it may be more efficient to consider the size and composition of species pools moved along particular pathways, rather than focusing on individual species (Brockerhoff et al., 2014). The greater the number of species introduced to a location (colonization pressure), the more species we should expect to establish self‐sustaining populations there (Blackburn et al., 2020; Lockwood et al., 2009). Similarly, the number of species transported via a given pathway or commodity is likely to be closely related to the introduction risk associated with such movement. While progress has been made toward understanding human‐mediated dispersal of certain taxa (e.g., Brockerhoff et al., 2006; Liebhold et al., 2012; Meurisse et al., 2019; Suarez et al., 2005; Ward et al., 2006), a global analysis of unintentional insect introduction pathways is lacking. Identifying commerce that transports a wide range of insects worldwide would improve our ability to monitor and manage key pathways, particularly in regions with fewer resources available.

The exact pathways responsible for historical species introductions are usually unknown, but alien species databases and inventories often assign species to the most likely pathway based on their ecology and the assumptions of the assessor (Essl et al., 2015; Kenis et al., 2007; Pergl et al., 2017). However, many countries perform inspections of trade goods, mail, and personal baggage at ports of entry (i.e., land borders, air and sea ports and transitional facilities) as a part of national biosecurity programs (Black & Bartlett, 2020; Saccaggi et al., 2016). Due to the large volume of trade, it is only possible to inspect a small fraction of imports (Natural Research Council, 2002). Therefore, inspections are typically not a primary method for excluding the arrival of potential pest species. However, inspection plays a key role in national biosecurity programs as a method for monitoring the presence of organisms in various pathways. This information is of great value in identifying invasion risks, setting phytosanitary policies (e.g., import bans and mandatory phytosanitary treatments) and monitoring compliance with existing import regulations (IPPC Secretariat, 2021; Sequeira & Griffin, 2014). Countries vary in their sampling methods, identification abilities, and the species and commodities they target (Turner et al., 2021; Whattam et al., 2014). Nonetheless, border interception records provide a unique window into the unintentional movement of insects and the commodities they are associated with.

In this study we combined interception records from six regions distributed across four continents to provide the first comprehensive overview of insect‐commodity associations in international trade and travel. Most studies of insect‐commodity associations have considered specific groups (e.g., taxa or feeding groups) of insects arriving in a single country, often on a preselected suite of commodities. Bark‐ and wood‐boring insects (e.g., Haack, 2006; Krishnankutty et al., 2020; Lawson et al., 2018; Liebhold et al., 2012; Messiner et al., 2008; Meurisse et al., 2019; Roques, 2010), agricultural pests (e.g., Areal et al., 2008; Caton et al., 2006; DeNitto et al., 2015; Kenis et al., 2007; McCullough et al., 2006; Smith et al., 2007) and ants (e.g., Lee et al., 2020; Suarez et al., 2001; Suhr et al., 2019; Ward et al., 2006; Yang et al., 2019) have been targeted in particular, probably due to the damage to forestry, agriculture and infrastructure that these taxa can cause (e.g., Aukema et al., 2011; Bradshaw et al., 2016; Jetter et al., 2002; Paini et al., 2016). In addition to using a standardized system for commodity classification, the broad taxonomic and geographic coverage of interceptions in this study could potentially improve efforts to make predictions about insect introduction pathways. Our aims are to: (1) quantify the richness and diversity of insect species transported with relevant commodities, (2) ascertain whether commodity associations vary among pathways (e.g., cargo vs. baggage vs. mail), (3) determine if key commodities vary among insect species, and groups of species, and (4) evaluate whether commodity associations are related to insect phylogeny.

## METHODS

### Data acquisition and cleaning

We analyzed the records of insects detected during inspections of international air and sea cargo, mail, vessels, and passenger baggage at ports of entry. The data consist of interceptions at air, land, and maritime ports from 1960 to 2019 in Australia, New Zealand, member countries of the European and Mediterranean Plant Protection Organisation (EPPO), Japan, USA, and Canada. As the number of individuals detected is not recorded in most regions, each interception represents a single arrival event per species. The insects discovered are destroyed, so although interceptions can be considered a proxy for species' unobserved arrival, they do not directly represent introductions. We included only interceptions between 1960 and 2019 for the years for which records were available in each region (Appendix S1: Table S1), and in which the insect was identified to the species level, with information available on the associated commodity. This timeframe corresponds to a period of increased globalization and trade openness (Baldwin & Martin, 1999; Feenstra et al., 2015; Klasing & Milionis, 2014). For most analyses, interceptions of genera with no members identified to species level were also included, as they represented at least one additional species.

We standardized insect taxonomic names across years and recording regions according to the Global Biodiversity Information Facility (GBIF) backbone taxonomy (GBIF Secretariat, 2019) using the *taxize* (Chamberlain & Szocs, 2013) and *rgbif* R packages (Chamberlain et al., 2021). GBIF has good coverage of insect taxonomy. Whereas the taxonomic names are not always the most recent, we prioritized standardizing to unique genus‐species names. The process was largely automated, but occasional unmatched species were corrected manually and a small proportion of synonyms may still be present.

We standardized commodity descriptions using the international Harmonized Commodity Description and Coding System (HS) for classifying traded goods (World Customs Organization, 2021) and subsequently grouped commodity codes into broad classes based on the type of product (Appendix S1: Figure S1). The HS is a hierarchical system of six‐digit codes, in which the first two digits (HS‐2) identify the chapter into which goods are classified (e.g., 08: Fruit and nuts, edible; peel of citrus fruit or melons). Some level of misclassification due to manual error may remain. Standardized classification based on HS codes provides commodity descriptions that can easily be integrated with trade data, and facilitates comparisons across countries and among studies. All analyses were conducted at the level of HS‐2 codes or broad commodity classes.

### Pooling data across interception regions

There are regional differences in inspection methods and targets, the sources, volume and nature of imports, and the years covered (Appendix S1: Table S1, Turner et al., 2021). However, the main commodity types associated with insects are similar across all regions, with the majority being plants, wood, and related products (Appendix S1: Figure S2). To test if species share similar commodity associations across regions despite the differences, we analyzed the 59 species intercepted more than 20 times in two or more regions. We included a separate commodity profile for each region in which a species was intercepted. We used a partial constrained correspondence analysis (CCA) in the *vegan* package (Oksanen et al., 2019) to estimate the variance in commodity associations explained by species, once the effect of interception region was removed. A CCA relates a matrix of species' abundance or occurrence to a matrix of explanatory variables. Partial CCA is an extension of this method in which the influence of conditioning variables in an additional matrix can be controlled (Legendre & Legendre, 2012). Pooling the interception records across countries allows us to analyze insect arrivals based on a much wider range of taxa and commodities, and to generalize across regions. As there was an overall similarity in the commodities recorded, and species shared similar commodity associations across regions, we pooled the data for further analysis.

### Estimating species richness and diversity

**FIGURE 1:**
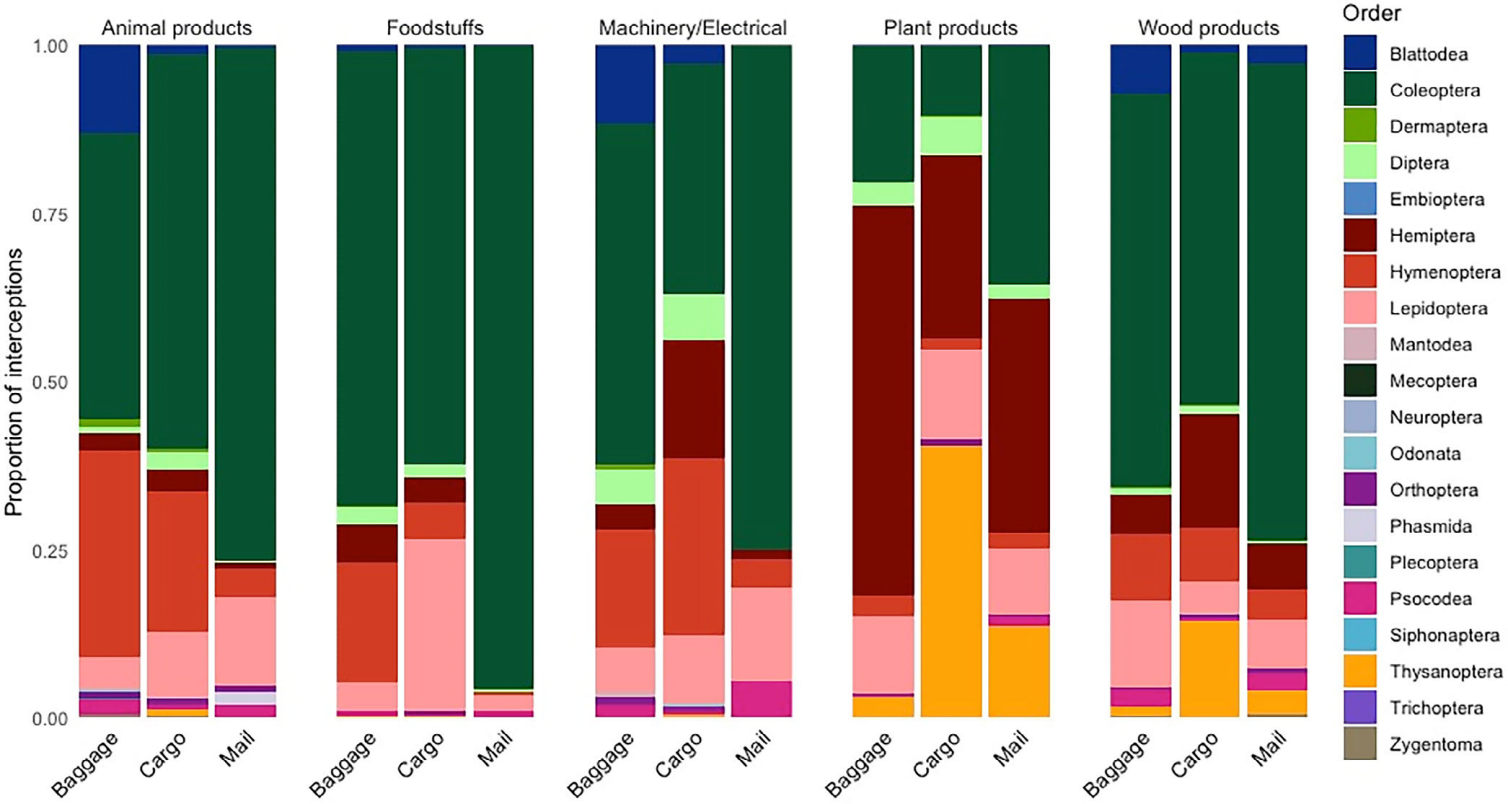
The taxonomic composition of interceptions with commodities arriving through the baggage, cargo, and mail pathways in Australia and the USA. The bars are colored by the proportion of interception events for each order. Only commodity classes with more than 20 interceptions in all three pathways are shown.

We used rarefaction and extrapolation methods to estimate total species richness (i.e., the number of species intercepted) and species diversity (i.e., the number and relative abundance of species) associated with different commodities, using the *iNEXT* package (Hsieh et al., 2016). The *ChaoRichness()* function estimates the asymptote of rarefaction and extrapolation curves and the associated confidence intervals based on the methods proposed in Chao (1984, 1987), giving a conservative lower bound for undetected species richness. Shannon's diversity index considers both the number of species (richness) and their relative abundance (evenness), which helps to distinguish between commodities in which species are transported with a similar frequency, and commodities in which only a few species are commonly intercepted. The *ChaoShannon()* function estimates Shannon diversity based on the method proposed by Chao et al. (2013). In addition to the commodity type, the pathway through which a commodity arrives is likely to influence which species have the opportunity to be transported. The relevant pathway was recorded for most interceptions in Australia and the USA. Only interceptions classed as cargo, passenger baggage, or international mail were comparable between the two countries. We estimated the species richness and Shannon diversity associated with commodities in each of these pathways as above. To compare the differences in taxonomic composition we carried out a PERMANOVA using the *adonis2()* function with Bray–Curtis distances in the *vegan* package (Oksanen et al., 2019) for orders intercepted with the five commodity classes found in all three pathways (Figure 1).

### Phylogenetic signal of commodity associations

Phylogenetic signal can be defined as the tendency for related species to resemble each other more than they resemble species drawn at random from the tree (Bloomberg & Garland Jr., 2002). To test whether related species shared similar commodity associations, we created a tree based on the taxonomic structure of species using the *as.phylo()* function in the *ape* package (Paradis & Schliep, 2018), adding branch lengths with the *compute.brlen()* function. We combined the taxonomic tree with each species' coordinates from the correspondence analysis (CA), and tested for phylogenetic signal using Abouheif's *C*_mean_ in the *phylosignal* package (Keck et al., 2016). The *C*_mean_ index was compared with the null hypothesis that the trait values are randomly distributed in the taxonomy (Keck et al., 2016). Molecular time estimates in Timetree.org (Kumar et al., 2017) represent a synthesis of published divergence time estimates (Hedges et al., 2015). We created an additional phylogenetic tree for the 150 species with available molecular time estimates (Appendix S1: Table S2), and tested for a phylogenetic signal to commodity associations as above.

We used three separate CCAs in the *vegan* package (Oksanen et al., 2019) to determine at what taxonomic level species share similar commodity associations, and the degree of variance explained by higher taxonomic levels. For each analysis of species “commodity profiles,” species' order, family, or genus was the single constraining variable. Taxa including only a single species were excluded from these analyses. The statistical significance of each model was assessed using a permutation test for CCA in the same package.

### Correspondence analysis and hierarchical clustering

To explore the relationship between species and the commodities with which they are transported, we carried out a CA using the *ade4* package (Dray & Dufour, 2007). We calculated the proportion of interceptions on each HS‐2 commodity group for each species, to compare their “commodity profiles” using the relative number of interceptions per commodity. Species with less than 20 interceptions were excluded, as this provided insufficient replication to characterize commodity associations. There were 1242 species intercepted a sufficient number of times for analysis. The first eight axes of the CA were retained. We used a hierarchical agglomerative clustering analysis in the *cluster* package (Maechler et al., 2019) to identify species associated with similar suites of commodities. Species were clustered based on their coordinates in the CA, using the *agnes()* function with Ward's clustering method (Kaufman & Rousseeuw, 1990). We used the permutation test introduced by Greenacre (2011) to determine whether nonrandom levels of clustering were present and, if so, to indicate at which level the resulting tree can be cut to give the optimal number of clusters. All analyses were conducted in R (R Core Team, 2017) and figures produced using the *ggplot2* package (Wickham, 2009).

## RESULTS

The dataset comprised 1,902,392 interception events, representing commodity associations for 7231 species and 1708 additional genera with no members identified to species level. The species intercepted were mainly Coleoptera (3165 species), Hemiptera (2708 species) and Lepidoptera (1103 species), but also included members of 19 other insect orders. Insects were intercepted on 80 different HS‐2 commodity groups, belonging to 14 different commodity classes (Appendix S1: Table S3). With the interception region included as a conditioning variable, species explained 46.7% of the variance in commodity associations, while the interception region explained just 12.3% of the variance in commodity associations. Both variables explained significantly more variance than expected by chance (permutation test for CCA with 999 permutations, interception region: *F* = 4.15, *p* = 0.001, species: *F* = 1.44, *p* = 0.001).

**FIGURE 2:**
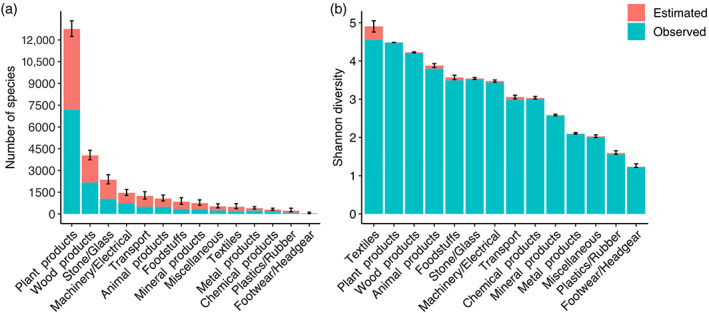
(a) The observed species richness (blue) and Chao1 estimates of additional undetected species richness (red) transported with each commodity class. (b) The observed (blue), and estimated additional undetected Shannon diversity (red) transported with each commodity class. The error bars indicate the standard error around the estimates of total richness and diversity.

**FIGURE 3:**
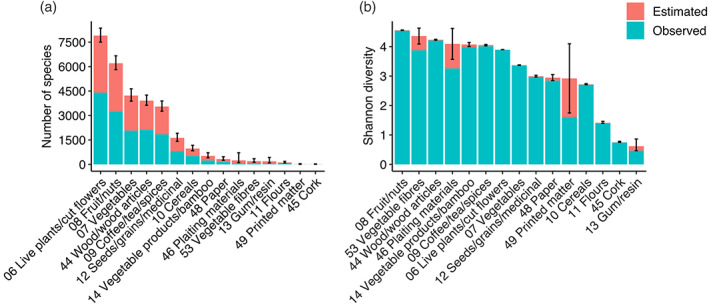
Interceptions with commodities classed as plant products or wood products. (a) The observed species richness (blue) and Chao1 estimates of additional undetected species richness (red) transported with each HS‐2 commodity group. (b) The observed (blue), and estimated additional undetected Shannon diversity (red) transported with each HS‐2 commodity group. The error bars indicate the standard error around the estimates of total richness and diversity.

Plant products (please refer to Table 1 for description of commodity groups) transported by far the most species, followed by wood products, stone and glass, and machinery and electricals. Textiles were associated with much lower species richness, but transported the highest insect diversity. Animal products and foodstuffs showed similar patterns (Figure 2). Within the broad categories of plant products and wood products, the HS‐2 commodities transporting the greatest species richness were live plants and cut flowers (HS 06), fruit and nuts (HS 08), vegetables (HS 07), wood and articles of wood (HS 44), and coffee, tea, herbs and spices (HS 09). Vegetable fibers (HS 53), plaiting materials (HS 46) and vegetable products and bamboo (HS 14) transported a high diversity of insects relative to species richness (Figure 3).

**TABLE 1:** Key commodity classes associated with insect movement, and the HS‐2 commodity groups belonging to each class

Commodity class	HS‐2 code	HS‐2 code full description according to the harmonized system
Animal products	01 Live animals	01 Animals; live
	02 Meat	02 Meat and edible meat offal
	03 Fish/crustaceans	03 Fish and crustaceans, molluscs and other aquatic invertebrates
	04 Dairy/eggs/honey	04 Dairy produce; birds' eggs; natural honey; edible products of animal origin, not elsewhere specified or included
	05 Animal products	05 Animal originated products; not elsewhere specified or included
	41 Hides/skins	41 Raw hides and skins (other than furskins) and leather
	42 Leather	42 Articles of leather; saddlery and harness; travel goods, handbags and similar containers; articles of animal gut (other than silk‐worm gut)
Plant products	06 Live plants/cut flowers	06 Trees and other plants, live; bulbs, roots and the like; cut flowers and ornamental foliage
	07 Vegetables	07 Vegetables and certain roots and tubers; edible
	08 Fruit/nuts	08 Fruit and nuts, edible; peel of citrus fruit or melons
	09 Coffee/tea/herbs/spices	09 Coffee, tea, mate and spices
	10 Cereals	10 Cereals
	11 Flours	11 Products of the milling industry; malt, starches, inulin, wheat gluten
	12 Seeds/grains/medicinal plants	12 Oil seeds and oleaginous fruits; miscellaneous grains, seeds and fruit, industrial or medicinal plants; straw and fodder
	13 Gum/resin	13 Lac; gums, resins and other vegetable saps and extracts
	14 Vegetable products and bamboo	14 Vegetable plaiting materials; vegetable products not elsewhere specified or included
	(1111) Soil around plants	(1111) Soil around plants
	53 Vegetable fibers	53 Vegetable textile fibers; paper yarn and woven fabrics of paper yarn
Foodstuffs	15 Oils/fats	15 Animal or vegetable fats and oils and their cleavage products; prepared animal fats; animal or vegetable waxes
	16 Meat/fish/crustacean preparations	16 Meat, fish or crustaceans, molluscs or other aquatic invertebrates; preparations thereof
	17 Sugars	17 Sugars and sugar confectionery
	18 Cocoa	18 Cocoa and cocoa preparations
	19 Cereal/flour preparations	19 Preparations of cereals, flour, starch or milk; pastrycooks' products
	20 Vegetable preparations	20 Preparations of vegetables, fruit, nuts or other parts of plants
	21 Food preparations	21 Miscellaneous edible preparations
	22 Beverages/vinegar	22 Beverages, spirits and vinegar
	23 Fodder/vegetable residue	23 Food industries, residues and wastes thereof; prepared animal fodder
	24 Tobacco	24 Tobacco and manufactured tobacco substitutes
Wood products	44 Wood/articles of wood	44 Wood and articles of wood; wood charcoal
	45 Cork	45 Cork and articles of cork
	46 Plaiting materials	46 Manufactures of straw, esparto or other plaiting materials; basketware and wickerwork
	47 Wood pulp	47 Pulp of wood or other fibrous cellulosic material; recovered (waste and scrap) paper or paperboard
	48 Paper	48 Paper and paperboard; articles of paper pulp, of paper or paperboard
	49 Printed matter	49 Printed books, newspapers, pictures and other products of the printing industry; manuscripts, typescripts and plans
Textiles	50 Silk	50 Silk
	51 Wool	51 Wool, fine or coarse animal hair; horsehair yarn and woven fabric
	52 Cotton	52 Cotton
	54 Synthetic fabric	54 Man‐made filaments; strip and the like of man‐made textile materials
	56 Twine/felt/rope/cables	56 Wadding, felt and nonwovens, special yarns; twine, cordage, ropes and cables and articles thereof
	57 Carpets	57 Carpets and other textile floor coverings
	61 Clothing, knitted	61 Apparel and clothing accessories; knitted or crocheted
	62 Clothing, not knitted	62 Apparel and clothing accessories; not knitted or crocheted
	63 Textile articles, tents	63 Textiles, made up articles; sets; worn clothing and worn textile articles; rags
Stone/Glass	68 Stone/plaster	68 Stone, plaster, cement, asbestos, mica or similar materials; articles thereof
	69 Ceramics	69 Ceramic products
	70 Glass	70 Glass and glassware
Machinery/Electrical	84 Machinery	84 Nuclear reactors, boilers, machinery and mechanical appliances; parts thereof
	85 Electricals	85 Electrical machinery and equipment and parts thereof; sound recorders and reproducers; television image and sound recorders and reproducers, parts and accessories of such articles

While plant products and wood products were associated with the highest richness and diversity across all three pathways (Appendix S1: Figure S5), there were some differences for HS‐2 commodities within these categories (Appendix S1: Figure S6). Wood and articles of wood (HS 44) transported the greatest number of species through mail, whereas in passenger baggage live plants and cut flowers (HS 06), wood and articles of wood (HS 44), fruit and nuts (HS 08), vegetables (HS 07) and coffee, tea, herbs and spices (HS 09) all transported high numbers of species (Appendix S1: Figure S6). These same commodities were important in cargo, with the most species associated with live plants and cut flowers (HS 06), and fruit and nuts (HS 08). Wood and articles of wood were associated with the greatest insect diversity in all three pathways (Appendix S1: Figure S6). The exact species intercepted on the same commodity types varied between cargo, baggage and mail (Appendix S1: Figure S4). However, whereas the commodity class had a significant effect on the taxonomic composition of insects (PERMANOVA with 9999 permutations, *F* = 2.48, *p* = 0.01), we found no significant effect of pathway (PERMANOVA with 9999 permutations, *F* = 0.58, *p* = 0.83).

Commodity associations were nonrandomly distributed among species, showing a phylogenetic signal both for the tree with relatedness based on taxonomy (Abouheif's *C*_mean_ 0.21–0.52, *p* = 0.001), and for the subset of species with information available on phylogenetic divergence times (Abouheif's *C*_mean_ 0.23–0.49, *p* = 0.001). The genus to which a species belonged explained 44.3% of the variance in species' commodity associations, whereas family explained 26.3% and order explained just 6.7% (please refer to Appendix S1: Table S4 for regional differences). All three taxonomic levels explained significantly more variance than expected by chance (permutation test for CCA with 999 permutations, genus: *F* = 2.47, *p* = 0.001, family: *F* = 3.61, *p* = 0.001, order: *F* = 9.64, *p* = 0.001).

**FIGURE 4:**
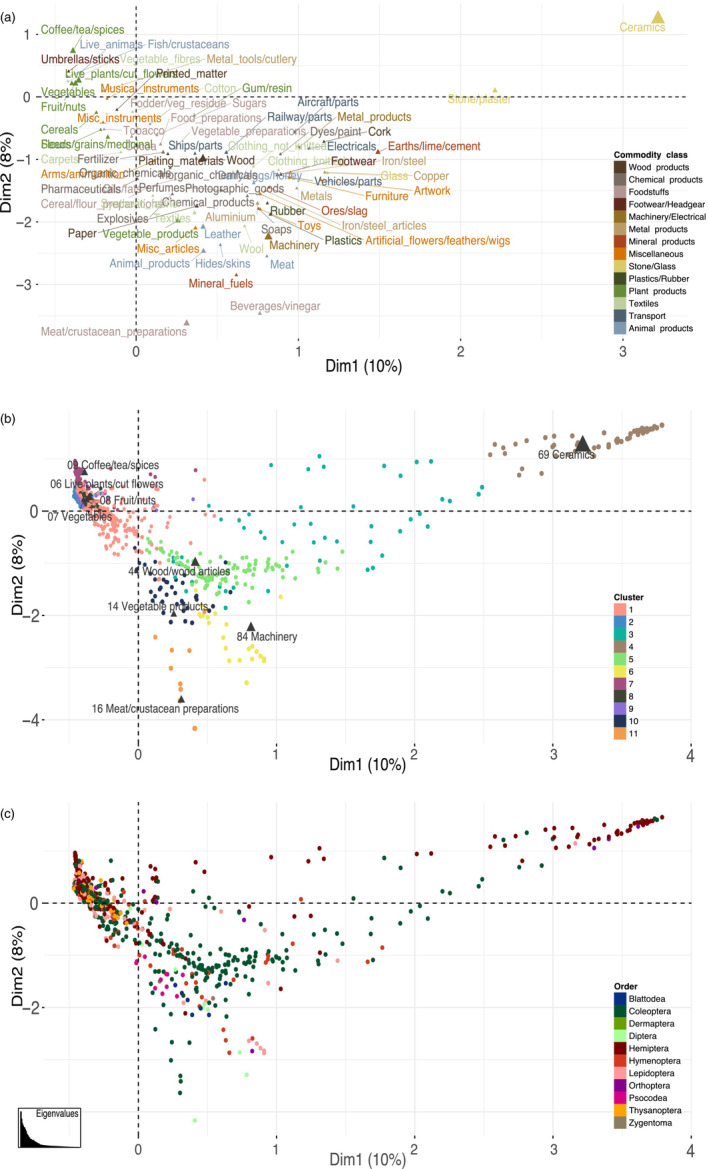
A correspondence analysis of species' commodity associations. (a) The HS‐2 commodity groups are colored by the broad commodity class to which they belong, the size of the triangles relates to their total contribution to the principal components. (b) Species are shown as circles colored by the cluster to which they belong, and the HS‐2 commodity groups on which they are most frequently intercepted is labeled. (c) Species are shown as circles colored by the order to which they belong.

We found 11 distinct clusters of species transported with similar suites of commodities (Figure 4). The first cluster consisted of 465 species most frequently intercepted with live plants and cut flowers (HS 06), but which were also frequently associated with fruit and nuts (HS 07). These species belong to the orders Hemiptera, Coleoptera, Lepidoptera, Thysanoptera, Hymenoptera, Diptera, Orthoptera, and Dermaptera, in decreasing order of species richness. The second cluster contained 64 species of Coleoptera, Lepidoptera, Hemiptera, Thysanoptera, Diptera, and Hymenoptera, which were most frequently intercepted with vegetables (HS 07). The third cluster was most often transported with ceramics (HS 69) and wood and articles of wood (HS 44), and consisted of 51 species of Coleoptera, Hymenoptera, Hemiptera, Lepidoptera, Blattodea, Orthoptera and Diptera. The fourth cluster of 53 species of Hemiptera, Coleoptera, Orthoptera and Lepidoptera were most frequently transported with ceramics (HS 69). The fifth cluster contained 107 species of Coleoptera, Hemiptera, Hymenoptera, and Lepidoptera which were mainly associated with wood and articles of wood (HS 44). The sixth cluster consisted of 23 species of Hymenoptera, Lepidoptera, Diptera, Coleoptera, Blattodea, Orthoptera, and Hemiptera, which were most frequently transported with machinery (HS 84). The seventh cluster consisted of 89 species most frequently transported with coffee, tea, herbs and spices (HS 09), belonging to the orders Hemiptera, Thysanoptera, Lepidoptera, Coleoptera, and Diptera. The eighth cluster consisted of 180 species of Hemiptera, Coleoptera, Thysanoptera, Lepidoptera, Diptera, Hymenoptera, and Dermaptera, which were most often associated with fruit and nuts (HS 08). The ninth cluster of 162 species were most frequently associated with live plants and cut flowers (HS 06), and belonged to Hemiptera, Coleoptera, Lepidoptera, Diptera, Orthoptera, Thysanoptera, Hymenoptera, and Blattodea. The 10th cluster consisted of 39 species of Coleoptera, Psocodea, Blattodea, Zygentoma, Hymenoptera, Lepidoptera and Hemiptera, and were most often intercepted with vegetable products and bamboo (HS 14). The 11th cluster consisted of just nine species of Coleoptera and Diptera, and was most frequently associated with meat and crustacean preparations (HS 16). Please refer to Appendix S1: Figure S3 for more details.

## DISCUSSION

The establishment of intentionally introduced organisms can be managed through regulations limiting importation and possession. However, prevention of unintentionally introduced organisms is more complex. It is first necessary to identify the major pathways by which these organisms are introduced, which individual national biosecurity agencies typically accomplish via pathway risk analyses (Essl et al., 2020; Hulme et al., 2008). We pooled border interception records spanning four continents to improve our knowledge of the commodities responsible for unintentional insect introductions. We found that plant and wood products were the dominant means of movement through international trade and travel. While this is well known for specific insect groups (e.g., Kiritani & Yamamura, 2003; Liebhold et al., 2012; Meurisse et al., 2019; Roques, 2010), our results highlight the wide range of taxa transported with these commodity types. Plant products and wood products were associated with the highest species richness in cargo, in international mail, and in passenger baggage, supporting their status as important targets for management across pathways. However, these were not the main commodities transporting all insect species, and many species were primarily associated with distinct commodity groups within these broad categories. This suggests that detailed information about relevant commodities is required for preventing the introduction of specific insect taxa.

The movement of plants and wood have long been recognized as important pathways for insect invasions (Kiritani & Yamamura, 2003; Liebhold et al., 2012; Meurisse et al., 2019; Roques, 2010). National biosecurity programs direct considerable effort toward limiting the accidental movement of insects through quarantine, inspection, mandatory phytosanitary treatments and other extensive preborder measures (Sequeira & Griffin, 2014), harmonized by the International Plant Protection Convention and other bodies (Hulme, 2011). We found that live plants and cut flowers, fruit and nuts, wood and articles of wood, vegetables, and coffee, tea, herbs and spices, in particular, transport a high number of species. While there is considerable variation in the insect taxa and commodity types considered in the literature, the importance of live plants (Liebhold et al., 2012; Eschen, Britton, et al., 2015; Meurisse et al., 2019), cut flowers (Areal et al., 2008; Hong et al., 2012; Kenis et al., 2007; Lee et al., 2016; McCullough et al., 2006; Roques & Auger‐Rozenberg, 2006; Suhr et al., 2019; Work et al., 2005), wood packaging material (Brockerhoff et al., 3^2006^; Haack, 2006; Krishnankutty et al., 2020; Lawson et al., 2018; Messiner et al., 2008), fruits and vegetables (Kenis et al., 2007; Lee et al., 2016; McCullough et al., 2006; Roques & Auger‐Rozenberg, 2006; Suhr et al., 2019; Work et al., 2005), and seeds (Franić et al., 2019; Kenis et al., 2007; McCullough et al., 2006) have been recognized previously. With the addition of coffee, tea, herbs, and spices as key plant products, our results support the idea that these commodities are major sources of insect introductions worldwide.

Whereas the same commodity types were generally important across pathways, the species richness and diversity associated with specific HS‐2 commodity groups varied (Appendix S1: Figure S6). The taxonomic composition of species associated with a commodity also differed between pathways, for example, proportionally more Hemiptera were associated with wood products in cargo than in baggage or mail. Commodities are often subject to different production and pest management practices depending on the pathway. Pathways also necessarily differ in the exact type, volume, treatment, and transport time of commodities, which in turn filters which species are present. For example, fresh fruits imported as commercial cargo typically undergo stringent care during production, and sometimes mandatory phytosanitary treatments to reduce pest risk. Fresh fruits arriving in baggage, conversely, may not have been commercially produced, and are controlled through inspection alongside public messaging. Pathway‐specific variation in pest management practices during the production, transport and arrival of commodities are likely to strongly influence which species are encountered during inspections.

The movement of textiles (Caton et al., 2006), and abiotic commodities such as machinery and building materials (McCullough et al., 2006), containers and used vehicles (Brockerhoff et al., 2006; Ward et al., 2006), and tiles (Haack, 2006; Work et al., 2005) have also been identified as important pathways for insect introductions. Ordination largely separated biotic commodities such as plant products and foodstuffs from wood products, and various abiotic commodities based on the associated species (Figure 4). The similarity in species associated with wood products and abiotic commodities may be due to the presence of wood packaging materials during transport. Up to 70% of all goods traded internationally (USDA cited in Eyre et al., 2018) are accompanied with some form of wood packaging, which offers a suitable substrate for many insect contaminants and hitchhikers. We are unable to distinguish between species transported with the packaging or the commodity itself based on the interception records, so the associated risk could also stem from the packaging. However, infestation rates of wood packaging materials are low (e.g., 0.17%–0.25% in the USA prior to ISPM15 [Haack et al., 2014]) and are unlikely to be a significant proportion of the records we assess here. We also found that textiles transport a particularly high diversity of insects relative to species richness, along with animal products and foodstuffs. It is likely that many species are only rarely associated with a given commodity and, due to the lower propagule pressure, will be less likely to establish (Kolar & Lodge, 2001; Lockwood et al., 2005). Commodities such as textiles in which species are more evenly transported may be sources of increased introduction risk.

However, a greater number of species introductions does not necessarily translate into greater impacts. National Plant Protection Organizations rely on species‐specific risk assessments to predict the potential damage caused by insects known to be associated with particular commodities. It should also be noted that, during the period from which we sourced data (1960–2019), there has been considerable progress in the implementation of new biosecurity practices that have probably reduced rates of commodity contamination and total numbers of species entering. For example, the harmonized international standard ISPM‐15 established by the International Plan Protection Convention specifies phytosanitary treatments for wood packaging, and has resulted in a substantial decrease in levels of wood‐boring insects present in this material (Haack et al., 2014). As another example, during this period the United States Department of Agriculture phased in new quarantine procedures for live plant imports that prohibit importation of plants of a large number of genera until risk analyses can be performed (USDA, 2021). Therefore, the numbers of species associated with commodities are likely to be changed during the period from which our data was sourced.

Prevention strategies that focus on high‐risk pathways alongside quarantine protocols targeting individual taxa are crucial for limiting the arrival of new and damaging species (e.g., Keller et al., 2009). Aichi Biodiversity Target 9 aimed that “by 2020, invasive alien species and pathways are identified and prioritized, priority species are controlled or eradicated, and measures are in place to manage pathways to prevent their introduction and establishment” (Convention on Biological Diversity, 2010). This clearly remains a work in progress (e.g., Tittensor et al., 2014), and continued research into pathway identification and management is necessary. Economic analyses are needed to evaluate whether the costs of additional biosecurity controls are smaller than the benefits of preventing invasions (Welsh et al., 2021). Moreover, future work could improve our estimates of species richness and diversity associated with different commodities by adjusting for import volume. The species contaminating or hitchhiking with a commodity are necessarily a subset of the species present in the region from which it originated, or potentially from intermediate stops along the way. Comparing the size and composition of species pools arriving from different world regions alongside associated trade volumes would help to further explain the patterns of introduction risk. We also observed that the degree of diversity in commodity associations varied considerably between taxa. Quantifying this variation would help to adjust the level of detail required for risk assessments and predictive modeling of different insect groups.

Species intercepted during port‐of‐entry inspections represent only a small proportion of the pool of insects arriving in a region (Kenis et al., 2007), and many species that arrive infrequently are likely to never be detected (Brockerhoff et al., 2014). The exact pathways of many new introductions are therefore unknown, and we may not have extensive knowledge about the commodities with which they are transported. On the condition that related insects tend to be transported with similar suites of commodities, species with known commodity associations could provide clues to the dispersal pathways of their more poorly observed relatives. Our results showed that related species do, to some extent, share similar commodity associations, although there remains much variation within insect taxa and interception regions. The similarities in commodity associations within genera could supply valuable information for targeting the pathway management of new species.

Interceptions provide direct evidence of an association between an organism and a commodity, but come with some limitations. Inspections often focus on commodities and pathways that have been previously considered high‐risk and may, preferentially or exclusively, record interceptions according to lists of regulated goods or regulated pest species (Eschen, Roques, et al., 2015). As the movement of plant and wood products are recognized as major pathways of insect introductions, they may be more frequently targeted for inspection. The greater intensity of inspections may therefore lead to more interceptions, irrespective of actual risk, creating a feedback to targeting these commodities. It is difficult to correct for inspection effort as practices vary between countries and pathways, and are adapted over time as risk assessments are updated, or new biosecurity policies come into force. Additionally, our analyses focus on records identified to the species level, and might not be representative of less easily identifiable taxa. Whereas our results are based on insects arriving in six different regions, these are high‐income countries and may not be representative of introductions to many developing nations. Unfortunately, negative inspections were not recorded. Randomized, statistically sound inspection systems such as the USDA Agricultural Quarantine Inspection Monitoring system (USDA, 2011) would provide greater power to quantify pathway risks when comparing and combining interception records, but are only focused on a few pathways in a few countries (Griffin, 2020).

The breadth and focus of inspections varies between regions, and alongside differences in import volume, production practices, trade partners, and biosecurity measures, are likely to influence the subset of commodity associations we observe (Saccaggi et al., 2016; Turner et al., 2021). In Europe, economically harmful plant‐pests are “black‐listed” from entering and being moved around the continent, and interceptions are largely restricted to these quarantine species (European Commission, 2002). Inspectors must check all consignments that could contain quarantine insects, but the exact sampling volumes and methods vary between the European member states (Bacon et al., 2012). Biosecurity programs in Australia and New Zealand operate based on “white‐lists” of species that have been assessed and are considered safe (Eschen, Britton, et al., 2015). However, from New Zealand we only had access to interceptions of ants (Formicidae) and forest insects, with a corresponding bias in associated commodities. In the USA, Canada, and Japan, the system is similar to Europe in that they have “black lists” of quarantine pests (Canadian Food Inspection Agency, 2021; US Department of Agriculture, Animal and Plant Health Inspection Service, 2020; Ministry of Agriculture and Fisheries, 2021), but these are generally less restrictive. Records from the USA made up the majority of both interception events and individual species intercepted, and our results were strongly influenced by the commodity associations of insects arriving in the USA (Appendix S1: Figure S7). Please refer to Appendix S1: Figures S8–S11 for more details about regional differences.

Nevertheless, the trends in commodity associations we observed are likely to be widely applicable. We used rarefaction and extrapolation methods to estimate species richness and diversity for standardized sample sizes (Chiarucci et al., 2008), so we expect the ranking of commodities to be robust. Although the list of commodities and species transported is almost certainly incomplete (Eschen, Roques, et al., 2015), the clusters of species associated with distinct commodities are likely to be robust. In most cases, inspection is not an effective method for excluding pest arrival and establishment directly, but provides crucial information for risk assessment. Pooling interception records across regions captures complementary aspects of the human‐mediated dispersal of insects, rather than focusing on insects arriving in a single region. The broad range of species and commodities intercepted provide a meaningful overview of the variation in commodity associations between and within taxa, as well as between pathways.

## CONCLUSIONS

Pathway analysis and management are powerful strategies for predicting and preventing new introductions of contaminant and hitchhiking insects. While knowledge of the exact pathways of unintentional introductions is scarce, pooling interception records across multiple regions provides a unique source of information on relevant commodities. Plant and wood products are important commodities across the cargo, baggage, and mail pathways. Live plants and cut flowers, fruit, and nuts, wood and articles of wood, vegetables, and coffee, tea, herbs, and spices, in particular, transport a high number of species. Commodities associated with high insect diversity, such as textiles, may be additional priorities for control measures.

Although plants, wood, and their associated products are important overall, the key targets for pathway management will not be the same for all alien species. Similarities in commodity associations within insect genera may provide valuable information for the management of potential previously unknown invaders. Our results highlight the wide range of commodities that are potential sources of new insect introductions, and the need for detailed information on relevant dispersal commodities to effectively limit future insect introductions.

## CONFLICT OF INTEREST

The authors declare no conflict of interest.

## Supporting information


Appendix S1

## Data Availability

Data (Fenn‐Moltu et al., 2022a) are available in Dryad at https://doi.org/10.5061/dryad.8931zcrrq. Code (Fenn‐Moltu et al., 2022b) is available in Zenodo at https://doi.org/10.5281/zenodo.6567146.
