# Supplementary material for: Alien insect dispersal mediated by the global movement of commodities
Source: Ecol Appl. 2022 Nov 13;33(1):e2721. doi: 10.1002/eap.2721 (PMC10078186; doi:10.1002/eap.2721)
Supplement: Supplementary file 1 — Appendix S1 [file EAP-33-0-s001.pdf]

Alien insect dispersal mediated by the global movement of commodities

Ecological Applications

Authors: Gyda Fenn-Moltu, Sébastien Ollier, Barney Caton, Andrew M. Liebhold, Helen Nahrung, Deepa S. Pureswaran, Rebecca M. Turner, Takehiko Yamanaka, Cleo Bertelsmeier

Supplementary material

Table S1: A description of the interception records available for each of the six regions.

Interception region	Data collection	Years covered	Total interception events	Orders	Species (+ additional genera)	% of species with > 20 interceptions	Commodity classes	HS-2 commodity groups	Source countries	Pathway information available
Australia	Department of Agriculture, Water and Environment	2003-2016	56955	19	1740	12.0 %	13	37	-	Yes
Canada	Canadian Food Inspection Agency	1997-2019	3165	12	926	2.4 %	9	29	89	No
European Plant Protection Organization (EPPO)	Various EPPO member countries	1995-2010	9464	7	303	10.2 %	2	5	116	No
Japan	Ministry of Agriculture, Forestry and Fisheries	1997-2017	289430	9	1235	18.9 %	6	14	128	No
New Zealand	Scion BUGS database, New Zealand Ministry for Primary Industries (or predecessors recorded under different names)	1960-2013 Formicidae<br><br>1960-2000 Forest insects	11759	3	553	12.7 %	14	62	122	No
United States of America	US Department of Agriculture, Animal & Plant Health Inspection Service, Department of Homeland Security, Customs and Border Protection	1998-2018<br><br>1960-2019 Formicidae<br><br>1960-1982 Thysanoptera<br><br>1960-2000 Forest insects	1531619	15	6827	12.9 %	14	78	220	Yes

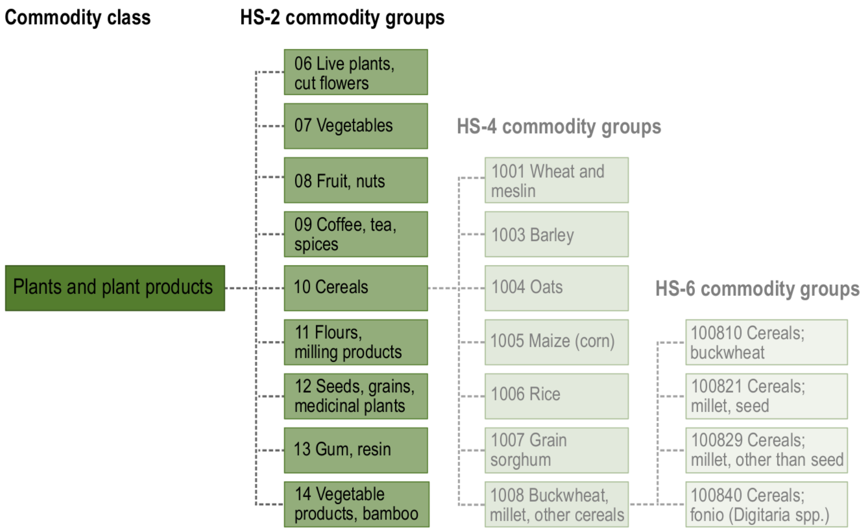

Figure S1: Plants and plant products as an example of the hierarchical classification of commodities. The first level of classification is based on the broad class of products, which is then further divided according to the international Harmonized Commodity Description and Coding Systems (HS) for classifying traded goods.

Table S2: the 150 species for which estimates of molecular divergence time were available from Timetree.org.

Species	Genus	Family	Order
Blatta orientalis	*Blatta*	Blattidae	Blattodea
Neostylopyga rhombifolia	*Neostylopyga*	Blattidae	Blattodea
Periplaneta americana	*Periplaneta*	Blattidae	Blattodea
Periplaneta australasiae	*Periplaneta*	Blattidae	Blattodea
Blattella germanica	*Blattella*	Ectobiidae	Blattodea
Supella longipalpa	*Supella*	Ectobiidae	Blattodea
Cryptotermes brevis	*Cryptotermes*	Kalotermitidae	Blattodea
Coptotermes formosanus	*Coptotermes*	Rhinotermitidae	Blattodea
Porotermes quadricollis	*Porotermes*	Stolotermitidae	Blattodea
Lyctus brunneus	*Lyctus*	Bostrichidae	Coleoptera
Cylas formicarius	*Cylas*	Brentidae	Coleoptera
Hylotrupes bajulus	*Hylotrupes*	Cerambycidae	Coleoptera
Acanthoscelides argillaceus	*Acanthoscelides*	Chrysomelidae	Coleoptera
Acanthoscelides obtectus	*Acanthoscelides*	Chrysomelidae	Coleoptera
Acanthoscelides obvelatus	*Acanthoscelides*	Chrysomelidae	Coleoptera
Bruchus pisorum	*Bruchus*	Chrysomelidae	Coleoptera
Callosobruchus chinensis	*Callosobruchus*	Chrysomelidae	Coleoptera
Callosobruchus maculatus	*Callosobruchus*	Chrysomelidae	Coleoptera
Callosobruchus phaseoli	*Callosobruchus*	Chrysomelidae	Coleoptera
Megabruchidius tonkineus	*Megabruchidius*	Chrysomelidae	Coleoptera
Stator limbatus	*Stator*	Chrysomelidae	Coleoptera
Necrobia rufipes	*Necrobia*	Cleridae	Coleoptera
Harmonia axyridis	*Harmonia*	Coccinellidae	Coleoptera
Dendroctonus pseudotsugae	*Dendroctonus*	Curculionidae	Coleoptera
Dinoplatypus pseudocupulatus	*Dinoplatypus*	Curculionidae	Coleoptera
Dryocoetes autographus	*Dryocoetes*	Curculionidae	Coleoptera

Hylurgops rugipennis	*Hylurgops*	Curculionidae	Coleoptera
Hylurgus ligniperda	*Hylurgus*	Curculionidae	Coleoptera
Hypera postica	*Hypera*	Curculionidae	Coleoptera
Platypus jansoni	*Platypus*	Curculionidae	Coleoptera
Tomicus piniperda	*Tomicus*	Curculionidae	Coleoptera
Xyleborus affinis	*Xyleborus*	Curculionidae	Coleoptera
Anthrenus verbasci	*Anthrenus*	Dermestidae	Coleoptera
Trogoderma granarium	*Trogoderma*	Dermestidae	Coleoptera
Trogoderma variabile	*Trogoderma*	Dermestidae	Coleoptera
Metamasius hemipterus	*Metamasius*	Dryophthoridae	Coleoptera
Sitophilus granarius	*Sitophilus*	Dryophthoridae	Coleoptera
Cryptolestes ferrugineus	*Cryptolestes*	Laemophloeidae	Coleoptera
Tenebrio molitor	*Tenebrio*	Tenebrionidae	Coleoptera
Tribolium castaneum	*Tribolium*	Tenebrionidae	Coleoptera
Tribolium confusum	*Tribolium*	Tenebrionidae	Coleoptera
Forficula auricularia	*Forficula*	Forficulidae	Dermaptera
Liriomyza bryoniae	*Liriomyza*	Agromyzidae	Diptera
Liriomyza huidobrensis	*Liriomyza*	Agromyzidae	Diptera
Liriomyza sativae	*Liriomyza*	Agromyzidae	Diptera
Liriomyza trifolii	*Liriomyza*	Agromyzidae	Diptera
Delia radicum	*Delia*	Anthomyiidae	Diptera
Chrysomya megacephala	*Chrysomya*	Calliphoridae	Diptera
Lucilia sericata	*Lucilia*	Calliphoridae	Diptera
Aedes aegypti	*Aedes*	Culicidae	Diptera
Aedes albopictus	*Aedes*	Culicidae	Diptera
Culex quinquefasciatus	*Culex*	Culicidae	Diptera
Drosophila melanogaster	*Drosophila*	Drosophilidae	Diptera
Atherigona orientalis	*Atherigona*	Muscidae	Diptera
Musca domestica	*Musca*	Muscidae	Diptera
Hermetia illucens	*Hermetia*	Stratiomyidae	Diptera
Anastrepha obliqua	*Anastrepha*	Tephritidae	Diptera
Bactrocera correcta	*Bactrocera*	Tephritidae	Diptera
Bactrocera cucurbitae	*Bactrocera*	Tephritidae	Diptera
Bactrocera dorsalis	*Bactrocera*	Tephritidae	Diptera
Bactrocera oleae	*Bactrocera*	Tephritidae	Diptera
Bactrocera zonata	*Bactrocera*	Tephritidae	Diptera
Ceratitis capitata	*Ceratitis*	Tephritidae	Dipt*era*
Dacus ciliatus	*Dacus*	Tephritidae	Diptera
Aleurodicus dispersus	*Aleurodicus*	Aleyrodidae	Hemiptera
Bemisia tabaci	*Bemisia*	Aleyrodidae	Hemiptera
Trialeurodes vaporariorum	*Trialeurodes*	Aleyrodidae	Hemiptera
Acyrthosiphon pisum	*Acyrthosiphon*	Aphididae	Hemiptera
Aphis aurantii	*Aphis*	Aphididae	Hemiptera
Aphis craccivora	*Aphis*	Aphididae	Hemiptera
Aphis fabae	*Aphis*	Aphididae	Hemiptera
Aphis gossypii	*Aphis*	Aphididae	Hemiptera
Aphis intybi	*Aphis*	Aphididae	Hemiptera
Aphis spiraecola	*Aphis*	Aphididae	Hemiptera
Brevicoryne brassicae	*Brevicoryne*	Aphididae	Hemiptera
Eriosoma lanigerum	*Eriosoma*	Aphididae	Hemiptera
Lipaphis pseudobrassicae	*Lipaphis*	Aphididae	Hemiptera
Myzus persicae	*Myzus*	Aphididae	Hemiptera
Rhopalosiphum maidis	*Rhopalosiphum*	Aphididae	Hemiptera
Rhopalosiphum nymphaeae	*Rhopalosiphum*	Aphididae	Hemiptera
Rhopalosiphum padi	*Rhopalosiphum*	Aphididae	Hemiptera
Tinocallis takachihoensis	*Tinocallis*	Aphididae	Hemiptera
Ceroplastes japonicus	*Ceroplastes*	Coccidae	Hemiptera
Coccus hesperidum	*Coccus*	Coccidae	Hemiptera
Aonidiella aurantii	*Aonidiella*	Diaspididae	Hemiptera
Parlatoria oleae	*Parlatoria*	Diaspididae	Hemiptera
Crypticerya genistae	*Crypticerya*	Margarodidae	Hemiptera
Icerya purchasi	*Icerya*	Margarodidae	Hemiptera
Icerya seychellarum	*Icerya*	Margarodidae	Hemiptera
Lygus rugulipennis	*Lygus*	Miridae	Hemiptera
Nesidiocoris tenuis	*Nesidiocoris*	Miridae	Hemiptera
Taylorilygus apicalis	*Taylorilygus*	Miridae	Hemiptera
Insignorthezia insignis	*Insignorthezia*	Ortheziidae	Hemiptera
Megacopta cribraria	*Megacopta*	Plataspidae	Hemiptera
Dysmicoccus brevipes	*Dysmicoccus*	Pseudococcidae	Hemiptera
Ferrisia virgata	*Ferrisia*	Pseudococcidae	Hemiptera
Planococcus citri	*Planococcus*	Pseudococcidae	Hemiptera
Pseudococcus longispinus	*Pseudococcus*	Pseudococcidae	Hemiptera
Pseudococcus maritimus	*Pseudococcus*	Pseudococcidae	Hemiptera
Pyrrhocoris apterus	*Pyrrhocoris*	Pyrrhocoridae	Hemiptera
Apis cerana	*Apis*	Apidae	Hymenoptera

Apis dorsata	*Apis*	Apidae	Hymenoptera
Apis mellifera	*Apis*	Apidae	Hymenoptera
Anoplolepis gracilipes	*Anoplolepis*	Formicidae	Hymenoptera
Camponotus pennsylvanicus	*Camponotus*	Formicidae	Hymenoptera
Cardiocondyla emeryi	*Cardiocondyla*	Formicidae	Hymenoptera
Crematogaster scutellaris	*Crematogaster*	Formicidae	Hymenoptera
Linepithema humile	*Linepithema*	Formicidae	Hymenoptera
Monomorium pharaonis	*Monomorium*	Formicidae	Hymenoptera
Ochetellus glaber	*Ochetellus*	Formicidae	Hymenoptera
Oecophylla smaragdina	*Oecophylla*	Formicidae	Hymenoptera
Paratrechina longicornis	*Paratrechina*	Formicidae	Hymenoptera
Solenopsis invicta	*Solenopsis*	Formicidae	Hymenoptera
Solenopsis molesta	*Solenopsis*	Formicidae	Hymenoptera
Solenopsis xyloni	*Solenopsis*	Formicidae	Hymenoptera
Tapinoma melanocephalum	*Tapinoma*	Formicidae	Hymenoptera
Tapinoma sessile	*Tapinoma*	Formicidae	Hymenoptera
Technomyrmex albipes	*Technomyrmex*	Formicidae	Hymenoptera
Tetramorium caespitum	*Tetramorium*	Formicidae	Hymenoptera
Wasmannia auropunctata	*Wasmannia*	Formicidae	Hymenoptera
Sirex noctilio	*Sirex*	Siricidae	Hymenoptera
Urocerus gigas	*Urocerus*	Siricidae	Hymenoptera
Xeris spectrum	*Xeris*	Siricidae	Hymenoptera
Estigmene acrea	*Estigmene*	Arctiidae	Lepidoptera
Lymantria dispar	*Lymantria*	Erebidae	Lepidoptera
Phthorimaea operculella	*Phthorimaea*	Gelechiidae	Lepidoptera
Lampides boeticus	*Lampides*	Lycaenidae	Lepidoptera
Agrotis ipsilon	*Agrotis*	Noctuidae	Lepidoptera
Mythimna unipuncta	*Mythimna*	Noctuidae	Lepidoptera
Spodoptera albula	*Spodoptera*	Noctuidae	Lepidoptera
Spodoptera cosmioides	*Spodoptera*	Noctuidae	Lepidoptera
Spodoptera dolichos	*Spodoptera*	Noctuidae	Lepidoptera
Spodoptera eridania	*Spodoptera*	Noctuidae	Lepidoptera
Spodoptera exigua	*Spodoptera*	Noctuidae	Lepidoptera
Spodoptera frugiperda	*Spodoptera*	Noctuidae	Lepidoptera
Spodoptera latifascia	*Spodoptera*	Noctuidae	Lepidoptera
Spodoptera littoralis	*Spodoptera*	Noctuidae	Lepidoptera
Spodoptera litura	*Spodoptera*	Noctuidae	Lepidoptera
Spodoptera ornithogalli	*Spodoptera*	Noctuidae	Lepidoptera
Trichoplusia ni	*Trichoplusia*	Noctuidae	Lepidoptera
Pieris rapae	*Pieris*	Pieridae	Lepidoptera
Hippotion celerio	*Hippotion*	Sphingidae	Lepidoptera
Macroglossum stellatarum	*Macroglossum*	Sphingidae	Lepidoptera
Cydia pomonella	*Cydia*	Tortricidae	Lepidoptera
Calliptamus italicus	*Calliptamus*	Acrididae	Orthoptera
Acheta domesticus	*Acheta*	Gryllidae	Orthoptera
Trogium pulsatorium	*Trogium*	Trogiidae	Psocodea
Frankliniella cephalica	*Frankliniella*	Thripidae	Thysanoptera
Thrips palmi	*Thrips*	Thripidae	Thysanoptera
Ctenolepisma longicaudata	*Ctenolepisma*	Lepismatidae	Zygentoma

Table S3: The commodity classes insects were intercepted on, the HS-2 commodity codes included in each class, and their descriptions according to the Harmonized System.

**Commodity class**	**HS-2 codes**	**HS-2 codes and full descriptions**
Animal products	01 Live animals, 02 Meat, 03 Fish/crustaceans, 04 Dairy/eggs/honey, 05 Animal products, 41 Hides/skins, 42 Leather	01 Animals; live, 02 Meat and edible meat offal, 03 Fish and crustaceans, molluscs and other aquatic invertebrates, 04 Dairy produce; birds' eggs; natural honey; edible products of animal origin, not elsewhere specified or included, 05 Animal originated products; not elsewhere specified or included, 41 Raw hides and skins (other than furskins) and leather, 42 Articles of leather; saddlery and harness; travel goods, handbags and similar containers; articles of animal gut (other than silk-worm gut)

Plant products	06 Live plants/cut flowers, 07 Vegetables, 08 Fruit/nuts, 09 Coffee/tea/herbs/spices, 10 Cereals, 11 Flours, 12 Seeds/grains/medicinal plants, 13 Gum/resin, 14 Vegetable products and bamboo, (1111) soil around plants, 53 Vegetable fibres	06 Trees and other plants, live; bulbs, roots and the like; cut flowers and ornamental foliage, 07 Vegetables and certain roots and tubers; edible, 08 Fruit and nuts, edible; peel of citrus fruit or melons, 09 Coffee, tea, mate and spices, 10 Cereals, 11 Products of the milling industry; malt, starches, inulin, wheat gluten, 12 Oil seeds and oleaginous fruits; miscellaneous grains, seeds and fruit, industrial or medicinal plants; straw and fodder, 13 Lac; gums, resins and other vegetable saps and extracts, 14 Vegetable plaiting materials; vegetable products not elsewhere specified or included, (1111) soil around plants, 53 Vegetable textile fibres; paper yarn and woven fabrics of paper yarn
Foodstuffs	15 Oils/fats, 16 Meat/fish/crustacean preparations, 17 Sugars, 18 Cocoa, 19 Cereal/flour preparations, 20 Vegetable preparations, 21 Food preparations, 22 Beverages/vinegar, 23 Fodder/vegetable residue, 24 Tobacco	15 Animal or vegetable fats and oils and their cleavage products; prepared animal fats; animal or vegetable waxes, 16 Meat, fish or crustaceans, molluscs or other aquatic invertebrates; preparations thereof, 17 Sugars and sugar confectionery, 18 Cocoa and cocoa preparations, 19 Preparations of cereals, flour, starch or milk; pastrycooks' products, 20 Preparations of vegetables, fruit, nuts or other parts of plants, 21 Miscellaneous edible preparations, 22 Beverages, spirits and vinegar, 23 Food industries, residues and wastes thereof; prepared animal fodder, 24 Tobacco and manufactured tobacco substitutes
Mineral products	25 Earths/lime/cement, 26 Ores/slag, 27 Mineral fuels	25 Salt; 5 Sulphur; earths, stone; plastering materials, lime and cement, 26 Ores, slag and ash, 27 Mineral fuels, mineral oils and products of their distillation; bituminous substances; mineral waxes
Chemical products	28 Inorganic chemicals, 29 Organic chemicals, 30 Pharmaceuticals, 31 Fertilizer, 32 Dyes/paint, 33 Perfumes, 34 Soaps, 36 Explosives	28 Inorganic chemicals; organic and inorganic compounds of precious metals; of rare earth metals, of radio-active elements and of isotopes, 29 Organic chemicals, 30 Pharmaceutical products, 31 Fertilizers, 32 Tanning or dyeing extracts; tannins and their derivatives; dyes, pigments and other colouring matter; paints, varnishes; putty, other mastics; inks, 33 Essential oils and resinoids; perfumery, cosmetic or toilet preparations, 34 Soap, organic surface-active agents; washing, lubricating, polishing or scouring preparations; artificial or prepared waxes, candles and similar articles, modelling pastes, dental waxes and dental preparations with a basis of plaster, 36 Explosives; pyrotechnic products; matches; pyrophoric alloys; certain combustible preparations
Plastics/Rubber	39 Plastics, 40 Rubber	39 Plastics and articles thereof, 40 Rubber and articles thereof
Wood products	44 Wood/articles of wood, 45 Cork, 46 Plaiting materials, 47 Wood pulp, 48 Paper, 49 Printed matter	44 Wood and articles of wood; wood charcoal, 45 Cork and articles of cork, 46 Manufactures of straw, esparto or other plaiting materials; basketware and wickerwork, 47 Pulp of wood or other fibrous cellulosic material; recovered (waste and scrap) paper or paperboard, 48 Paper and paperboard; articles of paper pulp, of paper or paperboard, 49 Printed books, newspapers, pictures and other products of the printing industry; manuscripts, typescripts and plans
Textiles	50 Silk, 51 Wool, 52 Cotton, 54 Synthetic fabric, 56 Twine/felt/rope/cables, 57 Carpets, 61 Clothing, knitted, 62 Clothing, not knitted, 63 Textile articles, tents	50 Silk, 51 Wool, fine or coarse animal hair; horsehair yarn and woven fabric, 52 Cotton, 54 Man-made filaments; strip and the like of man-made textile materials, 56 Wadding, felt and nonwovens, special yarns; twine, cordage, ropes and cables and articles thereof, 57 Carpets and other textile floor coverings, 61 Apparel and clothing accessories; knitted or crocheted, 62 Apparel and clothing accessories; not knitted or crocheted, 63 Textiles, made up articles; sets; worn clothing and worn textile articles; rags
Footwear/Headgear	64 Footwear, 66 Umbrellas/sticks	64 Footwear; gaiters and the like; parts of such articles, 66 Umbrellas, sun umbrellas, walking-sticks, seat sticks, whips, riding crops; and parts thereof
Stone/Glass	68 Stone/plaster, 69 Ceramics, 70 Glass	68 Stone, plaster, cement, asbestos, mica or similar materials; articles thereof, 69 Ceramic products, 70 Glass and glassware
Metal products	72 Iron/steel, 73 Iron/steel articles, 74 Copper, 75 Nickel, 76 Aluminium, 80 Tin, 81 Metals, 82 Metal tools/cutlery, 83 Metal products	72 Iron and steel, 73 Iron or steel articles, 74 Copper and articles thereof, 75 Nickel and articles thereof, 76 Aluminium and articles thereof, 80 Tin; articles thereof, 81 Metals; n.e.c., cermets and articles thereof, 82 Tools, implements, cutlery, spoons and forks, of base metal; parts thereof, of base metal, 83Metal; miscellaneous products of base metal
Machinery/Electrical	84 Machinery, 85 Electricals	84 Nuclear reactors, boilers, machinery and mechanical appliances; parts thereof, 85 Electrical machinery and equipment and parts thereof; sound recorders and reproducers; television image and sound recorders and reproducers, parts and accessories of such articles
Transport vectors and commodities	86 Railway/railway parts, 87 Vehicles/vehicle parts, 88 Aircraft/aircraft parts, 89 Ships/ships parts	86 Railway, tramway locomotives, rolling-stock and parts thereof; railway or tramway track fixtures and fittings and parts thereof; mechanical (including electro-mechanical) traffic signalling equipment of all kinds, 87 Vehicles; other than railway or tramway rolling stock, and parts and accessories thereof, 88 Aircraft, spacecraft and parts thereof, 89 Ships, boats and floating structures

Miscellaneous	67 Artificial flowers/feathers/wigs, 90 Miscellaneous instruments, 91 Clocks, 92 Musical instruments, 93 Arms/ammunition, 94 Furniture, 95 Toys, 96 Miscellaneous articles, 97 Artwork	67 Feathers and down, prepared; and articles made of feather or of down; artificial flowers; articles of human hair, 90 Optical, photographic, cinematographic, measuring, checking, medical or surgical instruments and apparatus; parts and accessories, 91 Clocks and watches and parts thereof, 92 Musical instruments; parts and accessories of such articles, 93 Arms and ammunition; parts and accessories thereof, 94 Furniture; bedding, mattresses, mattress supports, cushions and similar stuffed furnishings; lamps and lighting fittings, n.e.c.; illuminated signs, illuminated name-plates and the like; prefabricated buildings, 95 Toys, games and sports requisites; parts and accessories thereof, 96 Miscellaneous manufactured articles, 97 Works of art; collectors' pieces and antiques

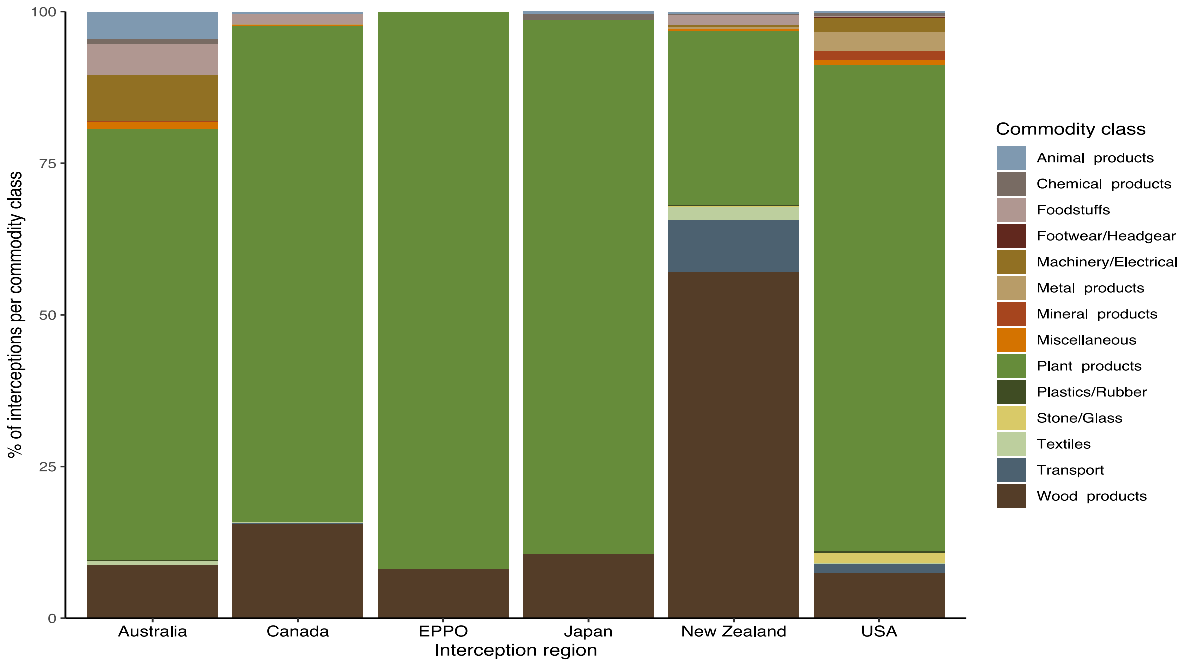

Figure S2: The proportion of interception events on each commodity class, in each of the six interception regions. The bars are coloured by the commodity class.

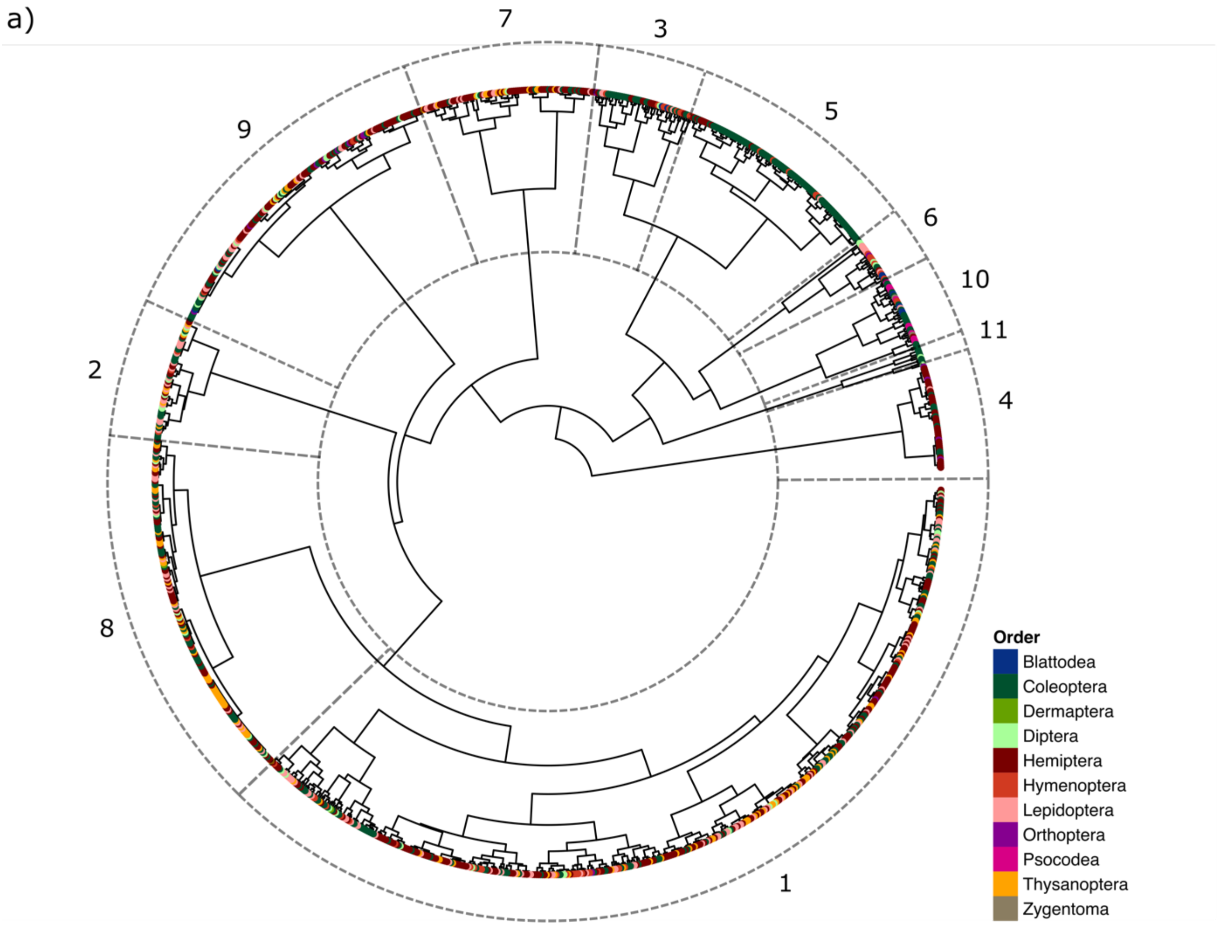

b)	1	2	3	4	5	6	7	8	9	10	11
Blattodea	0	0	2	0	0	2	0	0	2	4	0
Coleoptera	105	19	24	9	92	2	9	48	33	18	7
Dermaptera	1	0	0	0	0	0	0	1	0	0	0
Diptera	15	6	1	0	0	4	2	7	12	0	2
Hemiptera	185	11	10	39	9	1	55	54	78	1	0
Hymenoptera	27	3	10	0	5	7	0	3	4	2	0
Lepidoptera	73	18	3	1	1	6	11	21	18	1	0
Orthoptera	2	0	1	4	0	1	0	0	8	0	0
Psocodea	0	0	0	0	0	0	0	0	0	11	0
Thysanoptera	57	7	0	0	0	0	12	46	7	0	0
Zygentoma	0	0	0	0	0	0	0	0	0	2	0
Total	465	64	51	53	107	23	89	180	162	39	9

Figure S3: a) Hierarchical agglomerative clustering of species' commodity associations based on their coordinates in the CA. The leaves are coloured by the order each species belongs to, and the clusters are numbered and outlined with dashed lines. b) the number of species per order belonging to each cluster.

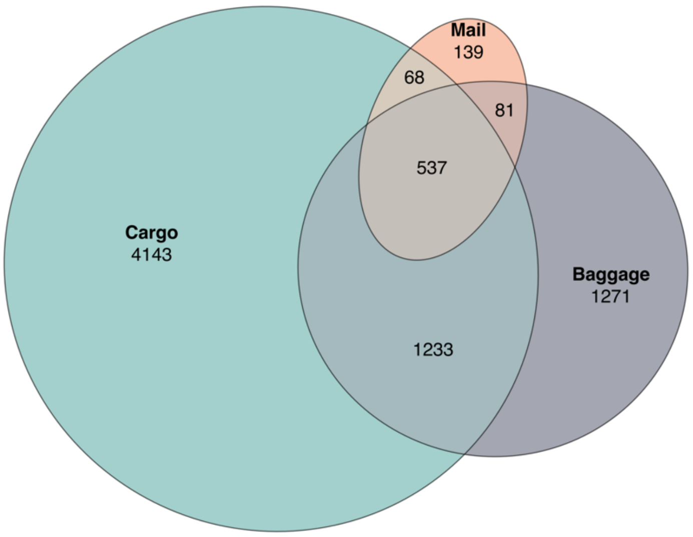

Figure S4: The number of species intercepted exclusively in cargo, in passenger baggage and in international mail, and the number of species intercepted in two, or all three pathways. The size of the circles is proportional to the number of species. The data is limited to the interceptions in Australia and the USA with information on the relevant pathway.

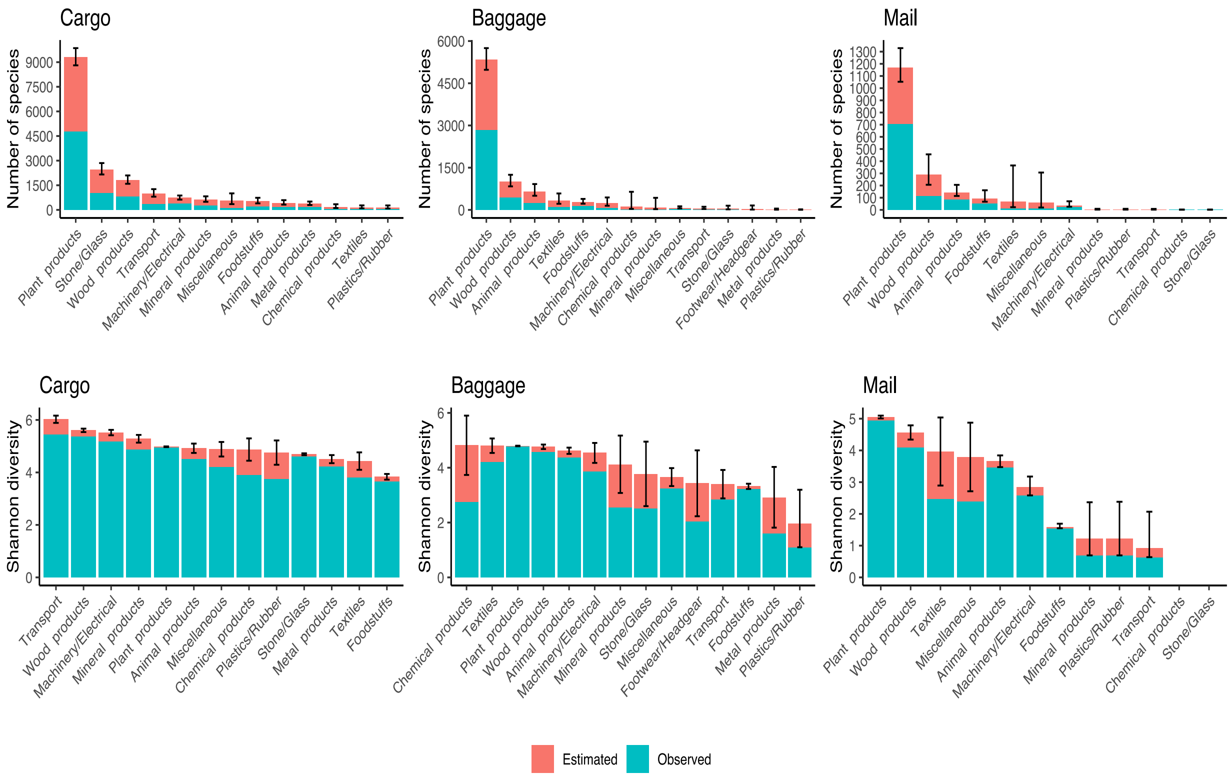

Figure S5: The observed (blue) and estimated (red) species richness and Shannon diversity

transported with each commodity class in cargo, baggage and mail. The data is limited to the interceptions in Australia and the USA with information on the relevant pathway.

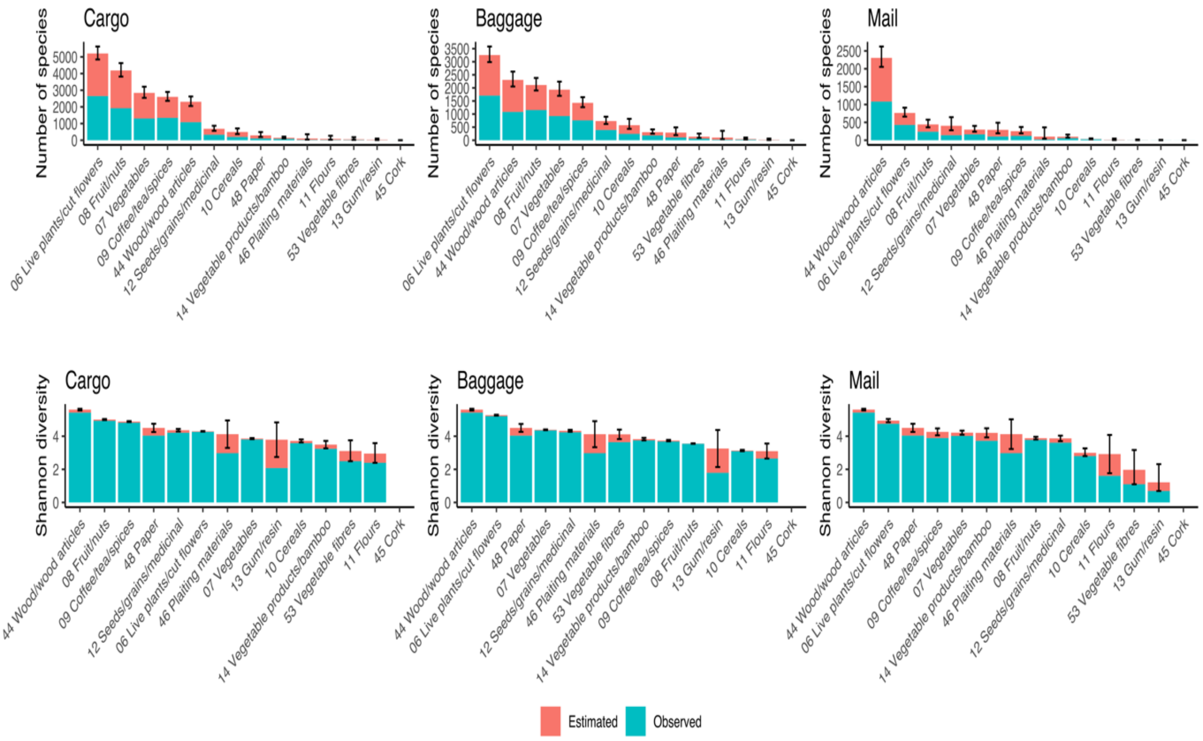

Figure S6: The observed (blue) and estimated (red) species richness and Shannon diversity transported with each HS-2 commodity group classed as plant products and wood products in cargo, baggage and mail. The data is limited to the interceptions in Australia and the USA with information on the relevant pathway.

Table S4: The percentage of variance in species' commodity associations explained by the order, the family and the genus they belong to, based on a series of CCAs (see methods section). P-values are adjusted for multiple hypothesis testing using the Bonferroni correction from the p.adjust() function in the stats package.

Interception region	Variance explained by order	Variance explained by family	Variance explained by genus
Australia	16.1 %, $p = 0.02$	52.2 %, $p = 0.02$	74.6 %, $p = 0.02$
Canada	24.3 %, $p = 0.94$	69.8 %, $p = 0.04$	-
EPPO	35.9 %, $p = 0.02$	59.6 %, $p = 0.02$	55.6 %, $p = 0.18$
Japan	3.9 %, $p = 0.68$	18.1 %, $p = 1$	42.4, $p = 0.02$
New Zealand	26.4 %, $p = 0.02$	32.5 %, $p = 0.02$	59.7 %, $p = 0.02$
United States	6.6 %, $p = 0.02$	25.6 %, $p = 0.02$	62.1 %, $p = 0.02$
Pooled	6.7 %, $p = 0.02$	26 %, $p = 0.02$	44 %, $p = 0.02$

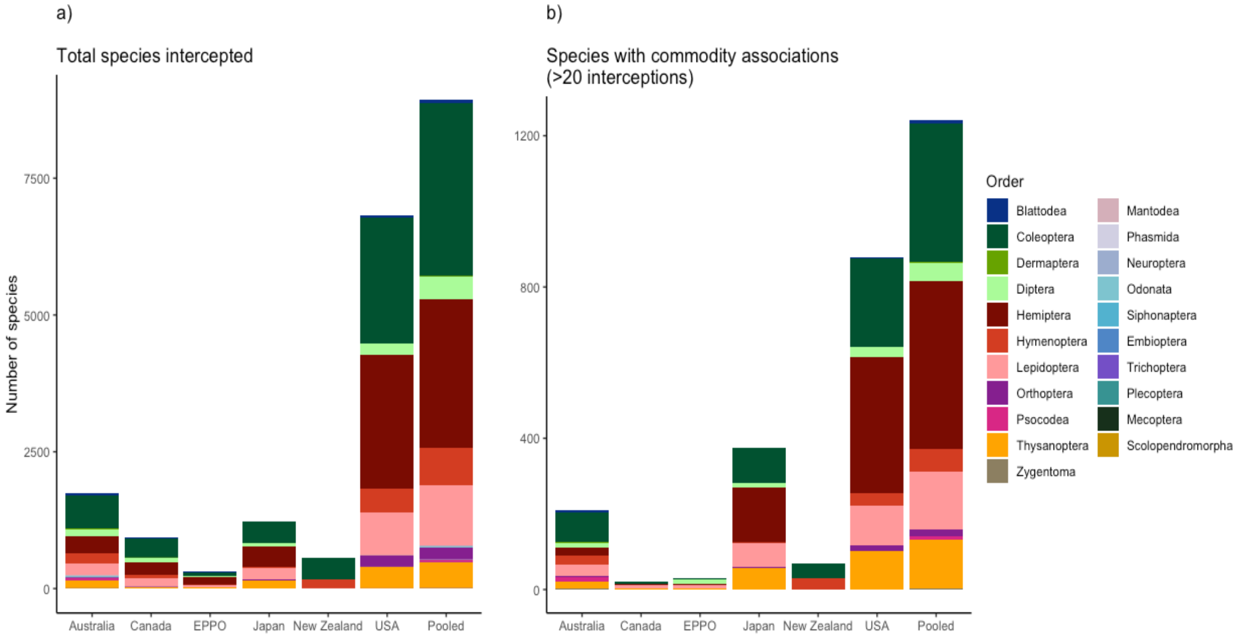

Figure S7: a) The total number of species, plus additional genera with no members identified to species level, intercepted in each region and in the pooled data, coloured by the order they belong to, and b) the number of species with more than 20 interceptions used to assess species' commodity associations in each region and in the pooled data, coloured by the order they belong to.

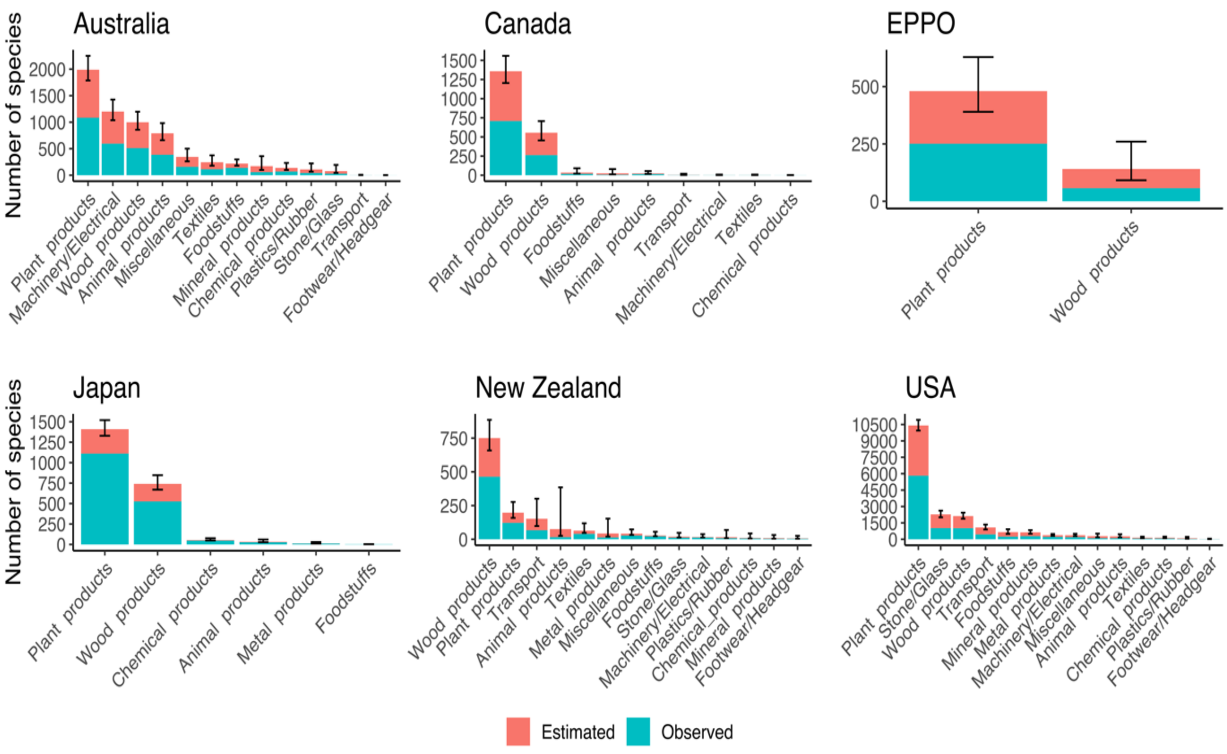

Figure S8: The observed species richness (blue) and Chao1 estimates of additional undetected species richness (red) transported with each commodity class, in each interception region. The error bars indicate the standard error around the estimates of total richness.

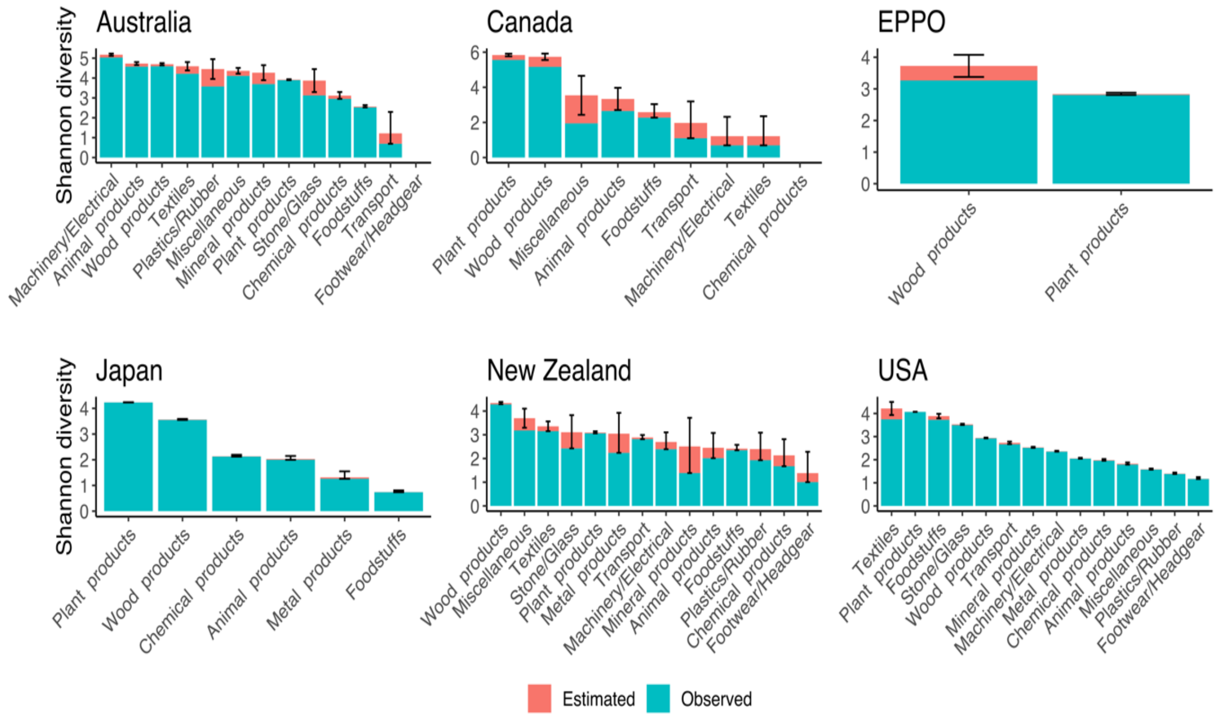

Figure S9: The observed Shannon diversity (blue) and Chao1 estimates of additional undetected diversity (red), transported with each commodity class, in each interception region. The error bars indicate the standard error around the estimates of total diversity.

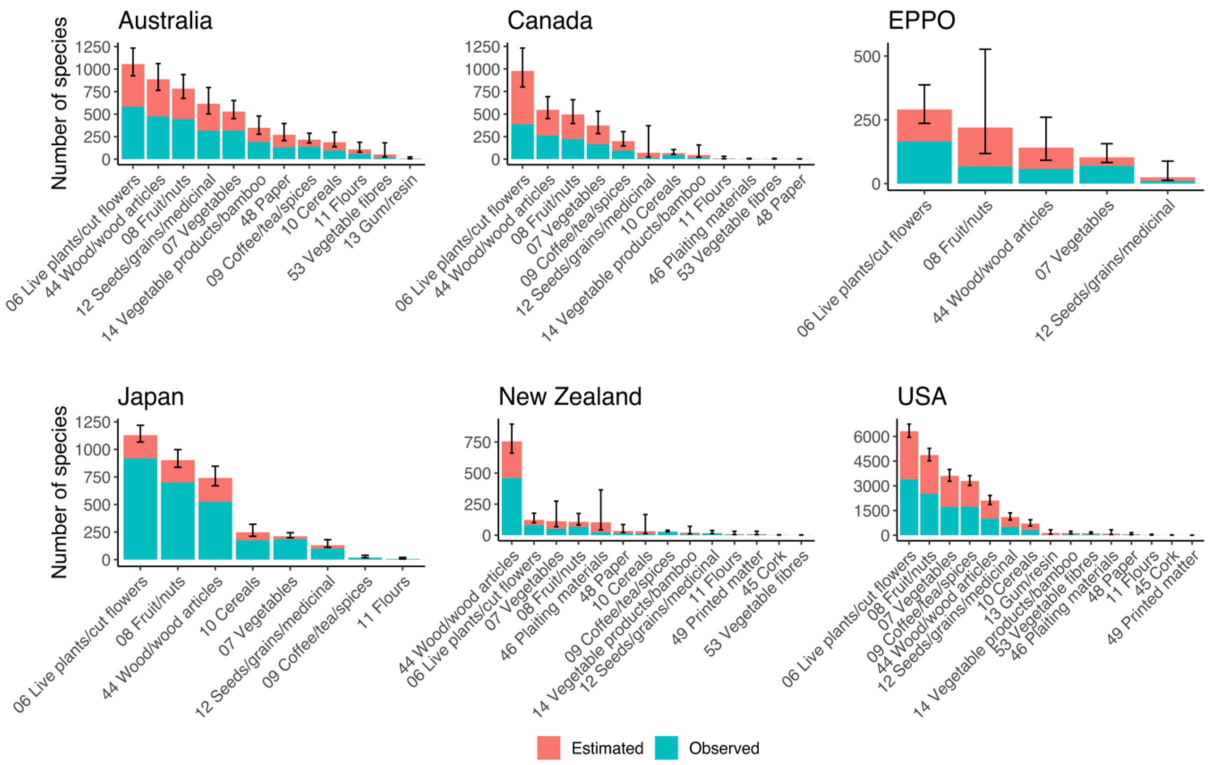

Figure S10: The observed species richness (blue) and Chao1 estimates of additional undetected species richness (red) transported with each HS-2 commodity group classed as plant products and wood products, in each interception region. The error bars indicate the standard error around the estimates of total richness.

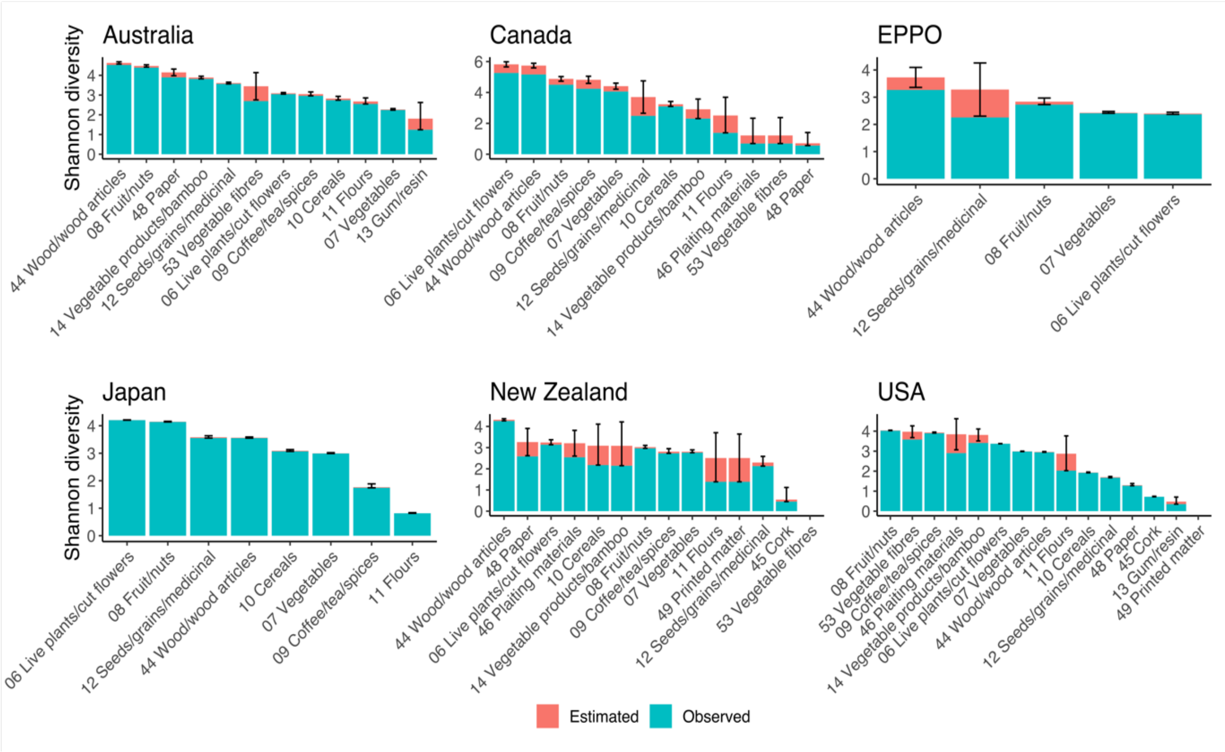

Figure S11: The observed Shannon diversity (blue) and Chao1 estimates of additional undetected diversity (red) transported with each HS-2 commodity group classed as plant products and wood products, in each interception region. The error bars indicate the standard error around the estimates of total diversity.
